# Lipid transfer to high‐density lipoproteins in coronary artery disease patients with and without previous cerebrovascular ischemic events

**DOI:** 10.1002/clc.23259

**Published:** 2019-09-06

**Authors:** Carlos J.D.G. Barbosa, Raul C. Maranhão, Renata S. Barreiros, Fatima R. Freitas, André Franci, Célia M.C. Strunz, Flávia B.B. Arantes, Thauany M. Tavoni, José A.F. Ramires, Roberto Kalil Filho, José C. Nicolau

**Affiliations:** ^1^ Hospital do Coracao do Brasil Brasília Brazil; ^2^ Instituto do Coracao (InCor), Hospital das Clinicas HCFMUSP, Faculdade de Medicina, Universidade de Sao Paulo Sao Paulo Brazil; ^3^ Faculdade de Ciencias Farmaceuticas, Universidade de Sao Paulo Sao Paulo Brazil; ^4^ Faculdade de Medicina da Universidade de Uberlandia Uberlandia Brazil

**Keywords:** CETP, coronary artery disease, high‐density lipoproteins (HDL), lipid transfers, stroke, transient ischemic attack

## Abstract

**Background:**

Patients with coronary artery disease (CAD) and previous ischemic cerebrovascular events (ICVE, ischemic stroke, or transitory ischemic attack) constitute a high‐risk subgroup for cardiovascular outcomes. High‐density lipoprotein cholesterol (HDL‐C) levels are correlated with cardiovascular events. Lipid transfer to HDL affects structure size and HDL subclass profile. Impairment of this transfer could influence ischemic risk seen in patients with CAD + ICVE. The objective was to evaluate the HDL ability to receive the lipids in patients with CAD with or without ICVE.

**Methods:**

Patients with CAD + ICVE (n = 60) and patients with CAD only (n = 60) were matched by age, sex, acute coronary syndromes (ACS) event type, and time elapsed between the ACS event and inclusion in the study. Lipid transfer to HDL was evaluated by incubating donor lipid nanoparticles labeled with radioactive unesterified cholesterol (UC) and esterified cholesterol (EC), phospholipid (PL), and triglyceride (TG) with whole plasma. After the chemical precipitation of non‐HDL fractions and nanoparticles, the supernatant was counted for HDL radioactivity.

**Results:**

CAD + ICVE group presented with impaired lipid transfer to HDL for PL (CAD + ICVE: 21.14 ± 2.7% vs CAD: 21.67 ± 3.1%, *P* = .03), TG (CAD + ICVE: 4.88 ± 0.97% vs CAD: 5.63 ± 0.92%, *P* = .002), and UC (CAD + ICVE: 5.55 ± 1.19% vs CAD: 6.16 ± 1.14%, *P* = .009). Lipid transfer to HDL was similar in both groups for EC. Adjusted models showed similar results.

**Conclusion:**

Patients with CAD and ICVE have reduced lipid transfer to HDL compared to those with CAD only. Dysfunctional HDL may account for the higher incidence of ischemic outcomes observed in this population.

## 1 INTRODUCTION

History of prior ischemic cerebrovascular event (ICVE), such as ischemic stroke or transitory ischemic attack, is reported in 2 to 17% of patients with coronary artery disease (CAD).^1^, ^2^, ^3^ This population of patients (CAD + ICVE) is of special concern because their risk for major cardiovascular events, including cardiovascular death, is significantly higher than those with a history of CAD only.^4^, ^5^

Observational studies suggest an inverse correlation between high‐density lipoprotein cholesterol (HDL‐C) levels and CAD, carotid atherosclerosis, myocardial infarction, stroke, and cardiovascular mortality.^6^, ^7^, ^8^, ^9^ HDL has a key role in cholesterol reverse transport, in which excess cholesterol from the peripheral tissues is shuttled back to the liver for excretion into the bile. Cholesterol esterification by lecithin–cholesterol acyltransferase (LCAT) occurs primarily in this lipoprotein fraction to stabilize the plasma cholesterol pool and drive reverse cholesterol transport.^10^ Several other protective functions have been attributed to HDL, such as antioxidant, anti‐inflammatory, antithrombotic, antiapoptotic, and vasodilation actions.^11^, ^12^

Lipid transfer to HDL is facilitated by cholesterol ester transfer protein (CETP) and phospholipid transfer protein (PLTP). This process affects the composition, structure, size and profile of HDL subclasses, the reverse cholesterol transport, and other protective functions of HDL.^13^, ^14^ Previous studies have shown that dysfunctional lipid transfer to HDL is related to cardiovascular risk factors and to CAD manifestations.^15^, ^16^, ^17^, ^18^

In this study, we hypothesized whether CAD + ICVE patients could show more pronouncedly altered rates of lipid transfers to HDL than CAD only patients. In this case, these alterations could contribute to the high risk of ischemic events in this population.

## 2 METHODS

### 2.1 Study design

This is a case–control study: the cases were patients with CAD and previous ICVE (CAD + ICVE group), and the controls were patients with CAD and without ICVE (CAD group). CAD was defined by the presence of coronary artery obstruction more than 50% during an acute coronary syndrome (ACS) event, and ICVE was defined by the presence of an ischemic stroke or transitory ischemic attack described as antecedent in medical record. From January 2013 to April 2015, the groups were selected retrospectively from two different institutional databanks and matched (1:1) for gender, age, previous ACS type (ST‐elevation myocardial infarction (STEMI), non‐ST‐elevation myocardial infarction (NSTEMI), and unstable angina), and time elapsed between the ACS episode and inclusion in the study. From this original population, we do not have data for 17 patients because of technical problems that precluded the lipid transfer to HDL analysis. Thus, after excluding incomplete pairs, 120 patients were included in the present analyses (60 and 60 in the CAD + ICVE and CAD groups, respectively).

Informed consent was obtained from each patient and the study was performed conforms to the ethical guidelines of the 1975 Declaration of Helsinki as reflected in a priori approval by the Ethics Committee of the University of São Paulo Medical School.

### 2.2 Inclusion criteria

Prior ACS, history of ischemic stroke or transitory ischemic attack prior to ACS, chronic use of aspirin, complete informed consent form, and complete evaluation of esterified cholesterol (EC), PL, triglyceride (TG), and unesterified cholesterol (UC) transfer to HDL.

### 2.3 Exclusion criteria

Prior hemorrhagic stroke, current dual antiplatelet therapy or anti‐inflammatory nonsteroidal therapy, thrombophilia or coagulopathy, thrombocytopenia (<1 000 000/mm^3^), thrombocytosis (>450 000/mm^3^), percutaneous coronary intervention or surgical myocardial revascularization in the last 6 months, severe renal impairment (CrCl<30 mL/min via the MDRD equation), and life expectancy <12 months.

### 2.4 Interventions

After a detailed review of the medical records and confirmation of the inclusion and exclusion criteria, patients were invited to participate. After signing the informed consent form, the patients underwent a clinical evaluation to confirm the data records and adherence to AAS.

### 2.5 Laboratory analyses

After 12 hours fasting, blood was collected to analyze blood cell counts, plasma lipid and glucose levels, and renal function parameters. Blood was also collected for evaluation of lipid transfer to HDL. The simultaneous transfer of EC, PL, TG, and UC from an artificial nanoparticle to HDL was measured in vitro, as described by Lo Prete et al.^19^ Briefly, donor lipid nanoparticle containing ^3^H‐cholesteryl oleate and ^14^C‐phosphatidylcholine or ^3^H‐triolein and ^14^C‐cholesterol was incubated with plasma under agitation for 1 hour at 37°C. After chemical precipitation of the nanoparticle and apo B‐containing lipoproteins, the supernatant containing the HDL fraction was counted for radioactivity in a scintillation solution. Then, the percentage of each lipid transferred from the nanoparticle to HDL was calculated.

### 2.6 Statistical analysis

The data are expressed as the mean and the SD, percentages and numbers, in the tables. At baseline, the mean values and percentages of occurrence between the groups were assessed by Student *t* test and *χ* ^2^ test, respectively.

Controls were propensity score matched to cases using age, sex, and time elapsed between the ACS, SBP, and us‐CRP, in order to exclude that few patients with high inflammation and oxidative stress. The procedure PSMATCH in SAS 9.4 was used with metric for similarity of observations that minimizes the difference between the logits of the propensity scores and greedy nearest neighbor for creating matched sets of observations. Unconditional logistic regressions were used to model the relationship between lipid transfers to HDL (EC, PL, TG, and UC) and presence or not of previous cerebrovascular ischemic events and compute odds ratios (ORs) with 95% confidence intervals. We use an unconditional analysis because there are no problems of sparse data and control for the matching factors can be obtained, with no loss of validity and a possible increase in precision and a “matched” (conditional) analysis may not be required or appropriate.^20^

Initially, univariate logistic regression analyses were conducted for clinical factors: weight, systolic blood pressure, diabetes, systemic arterial hypertension, use of statin, use of calcium channel blocker, number of antihypertensive medications, glycosylated hemoglobin, and ultrasensitive reactive C protein. Variables presenting *P* < .25 in the univariate analysis were included in the multivariate model. Subsequently, regression models were constructed by consecutive exclusion of variables from the complete model. Using the likelihood ratio test, we kept in the model factors with *P* < .05. Finally, lipid transfers to HDL (EC, PL, TG, and UC) were introduced separately into four multivariate models. Nonsignificant variables were retained in the model as confounders of removal resulted in an estimated change more than 15%, and *P* values <.05 were considered significant. The statistical analyses were performed using SAS software, version 9.4 (SAS Institute Inc. Cary).

## 3 RESULTS

### 3.1 Study population

Of the 120 patients included in the present analyses, 70% were males, with a mean age of 67 years; 71% had previous AMI, and the ACS index event occurred 5 years on average before study inclusion.

### 3.2 Baseline characteristics

As seen in Table 1, the case and control groups were adequately matched according to prespecified variables. However, patients in the CAD + ICVE group showed significantly higher SBP levels (*P* = .01), even though calcium channel blockers were more common in patients with CAD + ICVE (*P* = .004). Regarding other medications, the groups were well matched.

**Table 1:** Baseline characteristics

	CAD group (n = 60)	CAD + ICVE group (n = 60)	*P*‐value
Age (years ± SD)^a^	67 ± 9	66 ± 9	.49
Male (%)^a^	70	71	.86
AMI (%)^a^	71.7	69.8	.17
Time since ACS (years ± SD)^a^	5.6 ± 2.7	5.0 ± 2.7	.20
BMI (kg/m^2^ ± SD)	27.45 ± 4.45	27.84 ± 5.64	.85
SBP (mmHg ± SD)	123.62 ± 16.61	135.48 ± 19.22	.01
DBP (mmHg ± SD)	75.75 ± 9.3	77.39 ± 10.92	.30
Diabetes (%)	45	49	.51
Hypertension (%)	85	93	.48
Hypercholesterolemia (%)	80	69.8	.14
Current smoker (%)	5.71	5.71	1.00
Atrial fibrillation (%)	2.9	7.1	.17
PCI (%)	31.43	31.43	1.00
*Medications*			
Statin (%)	95	95.2	.95
Antihypertensive medications (n ± SD)	2.23 ± 1.06	2.89 ± 1.1	.08
ACE inhibitor (%)	57.14	54.29	.74
ARB (%)	15	27	.12
Calcium channel blocker (%)	20	44.4	.004
Diuretic (%)	35	42.9	.46
Beta blocker (%)	78.3	88.9	.14
Oral hypoglycemic medication (%)	45	41.3	.71
Insulin (%)	16.7	9.5	.29
Oral anticoagulant (%)	3.4	7.9	.16
*Metabolic and inflammatory tests*			
Total cholesterol (mg/dL)	166 ± 38	163 ± 39	.65
LDL‐C (mg/dL)	93 ± 32	92 ± 30	.87
HDL‐C (mg/dL)	44 ± 10	43 ± 13	.66
Triglyceride (mg/dL)	144 ± 96	133 ± 60	.36
BUN (mg/dL)	41.46 ± 15.17	44.26 ± 14.03	.26
GFR (mL/min/1.73 m^2^)	68.63 ± 22.1	65.5 ± 22.23	.28
HBA1c (%)	7.17 ± 2.27	7.66 ± 9.2	.68
Lipoprotein(a) (mg/dL)	45 ± 45	47 ± 44	.81
us‐CRP (mg/dL)	3.55 ± 4.32	6.48 ± 9.26	.13

a Prespecified matched variables.

Abbreviations: ACE, angiotensin converting enzyme; ACS, acute coronary syndromes; AMI, acute myocardial infarct; ARB, angiotensin receptor blocker; BMI, body mass index; BUN, blood urea nitrogen; DBP, diastolic blood pressure; GFR, glomerular filtration rate; HBA1c, glycated hemoglobin; HDL‐C, high‐density lipoprotein cholesterol; LDL‐C, low‐density lipoprotein‐cholesterol; PCI, percutaneous coronary intervention; SBP, systolic blood pressure; us‐CRP, ultrasensitive C‐reactive protein.

There were no statistically significant differences between the groups regarding risk factors for atherosclerosis and cardiovascular diseases. Moreover, all metabolic and inflammatory variables are similar among the groups.

### 3.3 Lipid transfer to HDL

**Figure 1:**
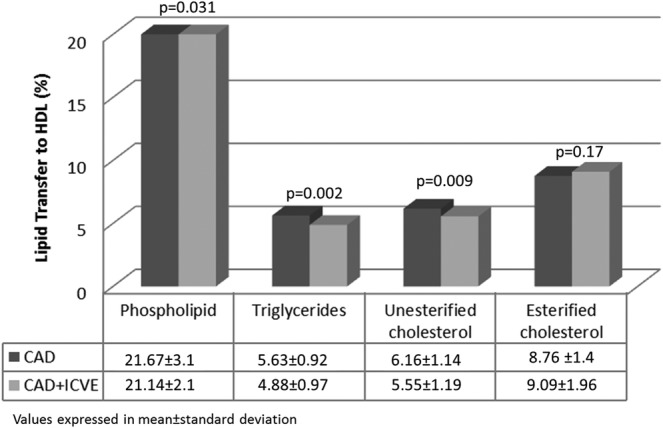
Comparison of lipid transfer to HDL between the groups

As shown in Figure 1, transfer of PL, TG, and UC to HDL was lower in the CAD + ICVE group than in the CAD group. EC transfer was not different between the two groups.

Table 2 presents results of the multivariable model. Using the cut off in the median values, the PL transfer to HDL below 24% implies in an adjusted OR of 7.64 for previous CAD + ICVE (*P* = .031), the TG transfer below 5.15% implies in an adjusted OR of 6.47 for previous CAD + ICVE (*P* = .004), and the UC transfer to HDL below 5% implies in an adjusted OR of 5.77 for previous CAD + ICVE (*P* = .011).

**Table 2:** Adjusted models with ischemic cerebrovascular event as dependent variable

	Unadjusted odds ratio	Adjusted odds ratio			
Lipid transfer to HDL (%)	OR (CI 95%)	*P*	OR (CI 95%)	*P*	
*Esterified cholesterol*		.1772		.2250
≤ 9.00	1	‐	1	‐
> 9.00	2.25 (0.69; 7.31)		2.35 (0.59; 9.19)	.2250
*Phospholipid*		.0317		.0306
≤ 24.00	4.00 (1.13; 14.17)	.0317	7.64 (1.21; 48.26)	.0306
> 24.00	1	‐	1	‐
*Triglyceride*		.0028		.0046
≤ 5.15	4.40 (1.67; 11.62)	.0028	6.47 (1.78; 23.53)	.0046
> 5.15	1	‐	1	‐
*Unesterified cholesterol*		.0092		.0113
≤ 5.00	4.25 (1.43; 12.63)	.0092	5.77 (1.49; 22.38)	.0113
> 5.00	1	‐	1	‐

Values dichotomized by median.

We performed a propensity score matching in order to exclude that few patients with high inflammation and oxidative stress might have affected the final results. The results of this analysis are according with the conditional logistic analysis (Supporting Information in Data S1).

## 4 DISCUSSION

The main finding of our study is that patients with CAD and previous ICVE have diminished lipid transfer to HDL compared to patients with only CAD. The occurrence of previous ischemic stroke or transitory ischemic attack in patients presenting with ischemic coronary syndromes represents an aggravated clinical condition. The risk for subsequent ischemic events is higher in these patients than in those without a history of prior cerebrovascular events.

In previous studies, it was shown that patients with CAD alone had lower UC transfer to HDL than individuals without CAD.^15^ Thus, the finding that UC transfer was further decreased in a group of patients with CAD + ICVE suggests that lipid transfer disturbances may also be linked to the worse outcomes in these populations.

Interestingly, lower rates of cholesterol transfer to HDL were previously associated with cardiovascular risk factors, such as type 2 diabetes^17^ and familial hypercholesterolemia.^16^ This feature is also present in patients with precocious coronary heart disease,^15^ but to the best of our knowledge, we are the first to describe an association with ischemic events in multiple vascular territories. The fact that the UC transfer rate was lower in CAD alone than in subjects without CAD and still lower in those with CAD + ICVE highlights the importance of lipid transfers not only as CAD risk factor but also as a possible indicator of aggravation and spreading of the atherosclerosis process to distant vascular beds. The in vitro method to evaluate lipid transfers to HDL, based on donor lipid emulsions, is straightforward and practical, and the adaptation to automatic procedures for the clinical laboratory analyses is clearly feasible. Future studies enrolling large populations are required to establish cut‐off points of normality and odds ratio estimations.

In respect to the potential importance of each lipid transfer to HDL in the clinical setting, it seems that the transfer of UC is the most physiologically meaningful, since the HDL fraction is the greatest cholesterol esterification plasma compartment and the entry of UC in this compartment at sufficient amounts is necessary. Lower rates of UC were also found not only in CAD patients but also in unhealthy conditions, such as sedentarism. In respect to PL and TG, the presumable clinical meaningfulness of the data is difficult to establish at this point of research.

The inverse correlation between HDL‐C and the incidence of cardiovascular disease has been well established in numerous studies of different populations.^6^, ^7^, ^9^, ^21^ However, many tested therapies failed to decrease cardiovascular events by improving HDL values.^21^, ^22^, ^23^, ^24^, ^25^, ^26^ A promising class of agents that increase HDL‐C by blocking CETP also failed to consistently demonstrate protective effects. One possible explanation for these disappointing outcomes is that the protective effects of HDL are dependent not only on higher HDL‐C levels but also on functional aspects of the lipoprotein that are not necessarily improved when HDL‐C is raised.^21^, ^27^, ^28^ Thus, altering the lipid transfers via CETP inhibitors could adversely affect the HDL action in reverse cholesterol transport and other HDL‐related protective actions, despite the marked cholesterol raising obtained with the use of CETP inhibitors.

Genetic aspects are probably related to HDL function, this is a possible explanation for individuals with similar values of HDL in which CETP inhibitor implies different clinical results for different genetic polymorphisms.^29^, ^30^ These polymorphisms are related with the cholesterol efflux in patients treated with dalcetrapib (CETP inhibitor); however, the relevance of genetics in transfer of lipids to HDL is unknown.^31^

Another situation that suggests the importance of function of HDL rather than blood concentration is present in several studies where individuals with very high HDL values present a higher cardiovascular risk than individuals with lower HDL values,^32^, ^33^, ^34^ revealing a possible atherothrombotic effect of dysfunctional HDL particles, as sugggest by our findings.

Hypertension is an important risk factor for ischemic and bleeding events.^35^ In our study, patients with CAD and ICVE had high SBP values, despite greater use of antihypertensive medications. These findings suggest that difficulty in controlling systolic hypertension in patients with ICVE could be related to the high risk of events in this population. In a mechanistic point of view, dysfunctional HDL may play a role in the development of arterial hypertension. Speer et al. showed that HDL promoted endothelial superoxide production, reduced nitric oxide bioavailability, and thus increased arterial blood pressure.^36^

Our study has some limitations. First, this is a retrospective design. Despite matched cases–controls for four different variables, other nonmatched variables could have influenced the results. Second, the case group had only mild sequels (mean modified Rankin Scale = 2), which may be related to the worse outcomes of patients with higher Rankin scores, precluding them to be included in the present study, no conclusion can be drawn for patients with a high degree of stroke sequels. Third, the absence of a healthy subject group precludes comparison with individuals without atherosclerosis. However, it has been described that patients with CAD have a lower transfer of lipids to HDL than individuals without CAD.^15^ It is remarkable that, while cholesterol efflux from THP‐1 monocytes to HDL, a well‐known test for HDL atheroprotective function was not associated with stroke events, as documented from MESA data,^37^ UC transfer to HDL measured here was even capable of telling apart subjects with CAD only from those with CAD and stroke.

## 5 CONCLUSION

In conclusion, our data indicate that patients with CAD and previous ICVE have lipid transfers to HDL more reduced than those with CAD only. Since transfer of UC to HDL has been consistently associated to CAD in previous studies, it is possible that impairment of the lipid transfer may play a role in the higher incidence of ischemic events in CAD patients with history of previous ICVE. Lipid transfers may also be markers of future CAD events in ICVE patients.

## CONFLICT OF INTEREST

The authors declare no potential conflict of interests.

## AUTHOR CONTRIBUTIONS

C.J.D.G.B. recruited the patients, performed the experiments, contributed to discussion, and wrote the manuscript. R.C.M. designed the study, contributed to discussion, and wrote the manuscript. R.S.B. recruited the patients, performed the experiments, and contributed to discussion. F.R.F. performed the experiments and contributed to discussion. A.F. performed the experiments and contributed to discussion. C.M.C.S. contributed to discussion. F.B.B.A. performed the experiments. J.A.F.R. contributed to discussion. R.K.F. contributed to discussion. J.C.N. designed and supervised the study, contributed to discussion, and wrote the manuscript. All the authors read and approved the final manuscript.

## Supporting information


**Data S1** Supporting Information
