# Supplementary material for: Lipid transfer to high‐density lipoproteins in coronary artery disease patients with and without previous cerebrovascular ischemic events
Source: Clin Cardiol. 2019 Sep 6;42(11):1100–5. doi: 10.1002/clc.23259 (PMC6837020; doi:10.1002/clc.23259)
Supplement: Supplementary file 1 — Data S1 Supporting Information [file CLC-42-1100-s001.doc]

**Suplementar Material**

Table 1 - Propensity score matching.

|  | **Odds Ratio (IC 95%)** | |
| --- | --- | --- |
|  | **Bivariada** | **p-valor** |
| **Weight** | 1.01 (0.98; 1.03) | 0.5788 |
| **Systemic arterial pressure** |  | 0.2203 |
| ≤ 120 | 1 | - |
| > 120 | 1.66 (0.74; 3.73) | 0.2203 |
| **DM** |  | 0.2998 |
| Não | 1 | - |
| Sim | 1.54 (0.68; 3.50) | 0.2998 |
| **HAS** |  | 0.1456 |
| Não | 1 | - |
| Sim | 2.54 (0.72; 8.89) | 0.1456 |
| **Statin use** |  | 1.0000 |
| Não | 1 | - |
| Sim | 1.00 (0.19; 5.22) | 1.0000 |
| **BloqCa use** |  | 0.0585 |
| Não | 1 | - |
| Sim | 2.28 (0.97; 5.35) | 0.0585 |
| **NumHAS** |  | 0.0421 |
| ≤ 2 | 1 | - |
| > 2 | 2.34 (1.03; 5.33) | 0.0421 |
| **ClCr** | 0.99 (0.98; 1.01) | 0.4602 |
| **usPCR** |  | 0.1038 |
| ≤ 2.35 | 1 | - |
| > 2.35 | 1.96 (0.87; 4.42) | 0.1038 |

DM, diabetes mellitus. HAS, systemic arterial hypertension. BloqCa, calcium channel blocker. NumHAS, number of antihypertensive medications. ClCr, creatinine clearance. usPCR, ultra-sensitive reactive C protein.

Table 2 - Adjusted models with myocardial infarction as dependent variable

|  | **Unadjusted Odds Ratio (OR)** | | **Adjusted Odds Ratio ^#^** | |
| --- | --- | --- | --- | --- |
| **Lipid transfer to HDL (%)^*^** | **OR (IC 95 %)** | **p-valor** | **OR (IC 95 %)** | **p-valor** |
| **Esterified cholesterol** |  | 0.7898 |  | 0.8213 |
| ≤ 9.00 | 1 | - | 1 | - |
| >9.00 | 1.12 (0.47; 2.67) | 0.7898 | 1.11 (0.45; 2.72) | 0.8213 |
| **Phospholipid** |  | 0.0258 |  | 0.0327 |
| ≤ 24.00 | 3.06 (1.14; 8.18) | 0.0258 | 3.02 (1.09; 8.32) | 0.0327 |
| > 24.00 | 1 | - | 1 | - |
| **Triglycerides** |  | 0.0059 |  | 0.0057 |
| ≤ 5.15 | 3.48 (1.43; 8.45) | 0.0059 | 3.73 (1.47; 9.48) | 0.0057 |
| >5.15 | 1 | - | 1 | - |
| **Unesterified cholesterol** |  | 0.0054 |  | 0.0042 |
| ≤ 5.00 | 9.10 (1.92; 43.14) | 0.0054 | 10.77 (2.12; 54.67 | 0.0042 |
| > 5.00 | 1 | - | 1 | - |

* Values ​​dichotomized by median.

# Adjusted by systemic blood pressure and use of calcium channel blocker
